# Photopic full-field electroretinography and optical coherence tomography in type 1 diabetic retinopathy

**DOI:** 10.1007/s00417-015-3034-y

**Published:** 2015-05-26

**Authors:** Ragnhild Wivestad Jansson, Maria Baroy Raeder, Jørgen Krohn

**Affiliations:** Department of Clinical Medicine, Section of Ophthalmology, University of Bergen, Bergen, Norway; Department of Ophthalmology, Haukeland University Hospital, 5021 Bergen, Norway; Department of Gynecology and Obstetrics, Haukeland University Hospital, Bergen, Norway

**Keywords:** Diabetes mellitus, Diabetic retinopathy, Fundus photography, Electroretinography, Optical coherence tomography, Retinal thickness

## Abstract

**Purpose:**

The purpose of this study was to evaluate the role of photopic full-field electroretinography (ERG) and retinal thickness measurements by spectral-domain optical coherence tomography (SD-OCT) in the assessment of disease severity in type 1 diabetic retinopathy.

**Methods:**

A population-based cohort of 151 patients with type 1 diabetes underwent a complete ophthalmic examination, including photopic full-field ERG and SD-OCT for retinal thickness measurements. Stereoscopic fundus photographs were taken according to the Early Treatment Diabetic Retinopathy Study protocol, and the classification of diabetic retinopathy was based on the International Clinical Diabetic Retinopathy Disease Severity Scale. Associations between photographically determined retinopathy level, b-wave amplitude and peak time of the photopic single-flash and 30-Hz flicker ERG, and central retinal thickness parameters were evaluated.

**Results:**

For all ERG measurements, the amplitude decreased and peak time increased with progression of the disease, but these associations lost statistical significance after adjusting for age and excluding laser-treated patients. Mean retinal thickness was significantly associated with the b-wave amplitude of photopic single-flash and 30-Hz flicker responses (r^2^ = 0.08, *p* = 0.006; and r^2^ = 0.05, *p* = 0.025, respectively), but revealed no association with retinopathy level.

**Conclusions:**

Photopic full-field ERG and SD-OCT-derived retinal thickness parameters have limited clinical value in the staging of diabetic retinopathy. However, thinning of the central retina leads to significant functional impairment and may reflect an ongoing neurodegenerative process in the retinal tissue.

## Introduction

The global prevalence of diabetes mellitus has been rapidly increasing, and it has become a disease of epidemic proportions [1]. Despite significant advances in medical and surgical management, diabetic retinopathy remains one of the most frequent and serious complications of diabetes. These trends necessitate early diagnosis and improved monitoring of ocular disease in order to optimize patient care and to reduce the burden of illness on patients and society. Assessment of retinal vascular changes, either by ophthalmoscopy or fundus photography, has traditionally been used to determine the severity of diabetic retinopathy [2–4]. However, several studies have shown that neuronal cell death, apoptosis, and retinal dysfunction may occur before microvascular disease can be detected [5, 6], and it is still unclear whether the retinal neuropathy is the result of diabetic microangiopathy, direct neurological damage from chronic hyperglycaemia, or both [7, 8].

Full-field electroretinography (ERG) is a non-invasive technique for evaluating global retinal function and is recognized as an important clinical tool for understanding the pathology of acquired retinal diseases [9, 10]. Full-field ERG studies in patients with diabetes have demonstrated a wide variety of changes associated with the severity of retinopathy [11]. One of the earliest signs of diabetic retinopathy, sometimes even preceding the vascular changes, is a reduction in the amplitude and an increase in the peak time (implicit time) of the oscillatory potentials [12, 13]. The summed amplitudes of the oscillatory potentials have been considered the most sensitive prognostic marker of progression to proliferative diabetic retinopathy [11, 14, 15]. A reduction in amplitude and delayed peak time of the b-wave in single-flash and 30-Hz flicker ERG have also been identified as significant markers of diabetic retinopathy, but are associated with more advanced disease [11, 16]. A few studies have evaluated photopic ERG responses in diabetic eyes. Holopigian et al. and Bresnick and Palta demonstrated that the peak time of photopic 30-Hz flicker ERG correlated significantly with the severity of diabetic retinopathy [17, 18]. Similarly, using photopic single-flash ERG, Satoh et al. found a delayed peak time and reduced b-wave amplitude, noting that peak time showed the highest association with progression of retinopathy [19].

Spectral domain optical coherence tomography (SD-OCT) is another non-invasive technique that has now become a standard method for diagnosing and monitoring diabetic macular oedema. Previous optical coherence tomography studies have shown inconsistent results regarding retinal morphological changes in diabetes patients, as some researchers have reported an increase [20–23] whereas others have reported a decrease in central retinal thickness [24–27]. Recent evidence suggests that selective thinning of the inner retinal layers takes place during the earliest stages of diabetic retinopathy, supporting the view that diabetic retinopathy is also a neurodegenerative disorder [7, 28].

Both photopic full-field ERG and SD-OCT are accurate, objective and readily available methods for assessing retinal function and morphology that may be useful in the clinical management of patients with diabetic retinopathy. However, results from previous studies are conflicting, study populations were heterogeneous, and most research has focused on differences between non-diabetic and diabetic subjects. Thus, the clinical value of different full-field ERG responses and retinal thickness measurements as markers of disease staging and progression are still unclear.

The main objectives of the present study were to examine the relationships between different stages of diabetic retinopathy assessed by fundus photography, various photopic full-field ERG parameters, and SD-OCT-derived retinal thickness measurements in a population-based cohort of patients with type 1 diabetes.

## Materials and methods

### Patients

Haukeland University Hospital is a tertiary care centre situated in the county of Hordaland, which has a population of about a half-million inhabitants (Statistics Norway 2013). Patients with type 1 diabetes mellitus were identified through a search, using the International Classification of Diseases (ICD) diagnosis codes for type 1 diabetes (E10 and all its subcategories), of the databases of hospitals, endocrinologists, and a selection of general practitioners in our region with a special interest in diabetes. The search identified 5545 patients, of whom a random sample of 1200 medical records were reviewed in order to exclude incorrectly diagnosed patients and those who were not residents of Hordaland County. This resulted in a randomly selected cohort of 350 patients with serological evidence of autoimmune type 1 diabetes. By the end of December 2013, 210 of these patients had been recruited for an ongoing epidemiological study on the prevalence and severity of diabetic retinopathy in Western Norway. Among these patients, those aged 17 years or older were asked to participate in the present ERG and SD-OCT study. Patients with retinal or optic nerve diseases other than diabetic retinopathy or media opacities leading to ungradable images and scans were excluded.

The study was registered and approved by the Regional Committee for Medical and Health Research Ethics, Western Norway, and followed the official ethical regulations for clinical research and the Declaration of Helsinki. All patients gave their written informed consent prior to participation in the study.

### Ophthalmologic examination, fundus photography, and classification of diabetic retinopathy

Information regarding patient demographics and medical history was collected from the medical records. All study participants underwent a detailed eye examination, with assessment of best-corrected visual acuity, measurement of intraocular pressure by Goldmann applanation tonometry, and slit lamp examination. Following pupil dilation with topical tropicamide (0.5 %) and phenylephrine hydrochloride (10 %), indirect ophthalmoscopy was performed with a 90-dioptre lens. Fundus photography was performed by an experienced ophthalmic photographer using the Canon CF-60Dsi digital fundus camera system combined with a Canon EOS-1D Mark II camera (Canon, Inc., Tokyo, Japan). The level of diabetic retinopathy was determined from stereoscopic colour fundus photographs with 40° fields of view that were taken of the seven standard fields according to the Early Treatment Diabetic Retinopathy Study (ETDRS) protocol [29]. In addition, a total of five red-free photographs with 60° fields of view were taken, as follows: centred on the macula, temporal to the macula, nasal to the optic disc, superior to the optic disc, and inferior to the optic disc. The colour fundus images were displayed on a calibrated LCD monitor and assessed with a handheld stereoscopic viewer (ScreenScope; Stereo Aids, Albany, Australia). All images were reviewed and graded by two retina specialists (RJ and JK), who were masked to other patient information. The level of diabetic retinopathy was graded according to the International Clinical Diabetic Retinopathy Disease Severity Scale: 1 = no retinopathy; 2 = mild non-proliferative retinopathy; 3 = moderate non-proliferative retinopathy; 4 = severe non-proliferative retinopathy; 5 = proliferative retinopathy [4]. Levels 1, 2, and 3 represent relatively low risk of visual loss, while levels 4 and 5 represent significant risk [4]. In the case of discrepancy, the fundus images were discussed by the two graders until mutual agreement was reached. Macular oedema was classified as either present or absent based on the presence of retinal thickening or hard exudates in the posterior pole [4].

### Electroretinography

After pharmacological pupil dilatation, light-adapted (photopic) full-field electroretinograms were recorded in both eyes using a Nicolet Viking IV D analysis system (Nicolet Biomedical Instruments, Madison, WI, USA). A Burian–Allen bipolar contact lens electrode was applied on the topically anaesthetized cornea (oxybuprocaine hydrochloride 0.4 %) and a ground electrode was placed on the patient’s forehead. Recordings were made in accordance with the standards of the International Society for Clinical Electrophysiology of Vision (ISCEV) [30]. Photopic responses (background illumination intensity = 34 cd/m^2^) were obtained with a wide-band filter (−3 dB at 1 Hz and 500 Hz) stimulating with single full-field flashes of white light (integrated luminance 3.93 cd.s/m^2^), and cone responses were obtained with 30-Hz flickering white light (integrated luminance 0.81 cd.s/m^2^) averaged from 20 sweeps. Recordings of both ERG measurements were repeated until two identical curves were obtained in order to ensure reproducibility. The b-wave amplitude and peak time were measured in both photopic single-flash and 30-Hz flicker ERG.

### Optical coherence tomography

All patients underwent SD-OCT imaging in both eyes through dilated pupils using the spectral domain Topcon 3D OCT-1000 Mark II (software version 3.51; Topcon Corp., Tokyo, Japan). SD-OCT scanning was performed by an experienced ophthalmic photographer. The protocol used for all eyes was a 3D macula scan based on a 6 × 6 mm rectangular scan area and 512 × 128 lines (128 horizontal scan lines comprising 512 A-scans), and an axial resolution of 6 μm. The central foveal thickness, mean retinal thickness, and total macular volume corresponding to the nine subfields of the 6-mm-diameter ETDRS macular grid were calculated [31]. Eyes with decentred SD-OCT scans were excluded.

### Statistical analysis

Two sets of analyses were conducted, one including and one excluding patients who had previously received laser treatment for diabetic retinopathy. We used one-way analysis of variance (ANOVA) statistics to evaluate the association between the five levels of retinopathy and the ERG and SD-OCT findings. Post hoc ANOVA testing was performed using Bonferroni’s multiple comparison test. The data were adjusted for age by analysis of covariance (ANCOVA). Simple linear regression was used to model the associations between ERG and SD-OCT measurements. The upper and lower limits of normal values were defined as the mean plus and minus two standard deviations, respectively. Comparison of two means was performed using the unpaired two-tailed *t* test. The level of interobserver agreement was estimated by Cohen’s kappa statistics. The data were analysed using Stata version 13.0 software (StataCorp LP, College Station, TX, USA). For all tests, two-tailed *p* values < 0.05 were considered statistically significant.

## Results

### Patients and fundus photography

The majority of the patients included in the epidemiologic study of diabetic retinopathy in Western Norway agreed to participate in the present ERG and SD-OCT study. Thus the study was performed in a randomly selected, population-based cohort of mainly adult patients with type 1 diabetes from the western part of Norway. The study included a total of 151 patients, of whom 71(47 %) were women and 80 (53 %) were men. The median age at the time of examination was 36 years (range, 17–74 years). One of the seven patients with severe non-proliferative diabetic retinopathy (level 4) and 18 of the 20 patients classified as having proliferative diabetic retinopathy (level 5) had previously undergone panretinal photocoagulation in both eyes. Patient demographics according to the levels of retinopathy are presented in Table 1.

**Table 1:** Patient demographics according to severity level of diabetic retinopathy

Variable	All patients	Patients grouped by level of diabetic retinopathy				
		1	2	3	4	5
Females, *n* (%)	71 (47)	28 (51)	20 (44)	11 (46)	2 (29)	10 (50)
Males, *n* (%)	80 (53)	27 (49)	25 (56)	13 (54)	5 (71)	10 (50)
Age at examination, years, median (range)	36 (17–74)	30 (17–59)	36 (19–58)	41 (21–74)	39 (31–54)	46 (34–70)
Duration of diabetes, years, median (range)	17 (0–63)	10 (0–38)	17 (4–46)	23 (10–63)	29 (21–34)	33 (18–60)

Photographically determined macular oedema was present in nine (6 %) patients (three female, six male) with a median age of 34 years (range, 21–49 years). Two patients had level 2, three had level 3, two had level 4, and two had level 5 diabetic retinopathy. In the majority of these patients, focal retinal thickening and hard exudates were seen in the parafoveal or extrafoveal regions. The patients with macular oedema had a mean central foveal thickness of 236 μm (range, 207–290 μm) and mean retinal thickness of 267 μm (range, 242–307 μm). Compared to the rest of the study population, they had significantly greater central foveal thickness (*p* = 0.02), while there was no significant difference in mean retinal thickness (*p* = 0.82).

The colour and red-free fundus photographs were of good diagnostic quality, allowing assessment of the level of diabetic retinopathy with a high degree of accuracy. The Cohen’s kappa value for agreement between the two graders was 0.92.

### Association between ERG parameters and level of diabetic retinopathy

**Fig. 1:**
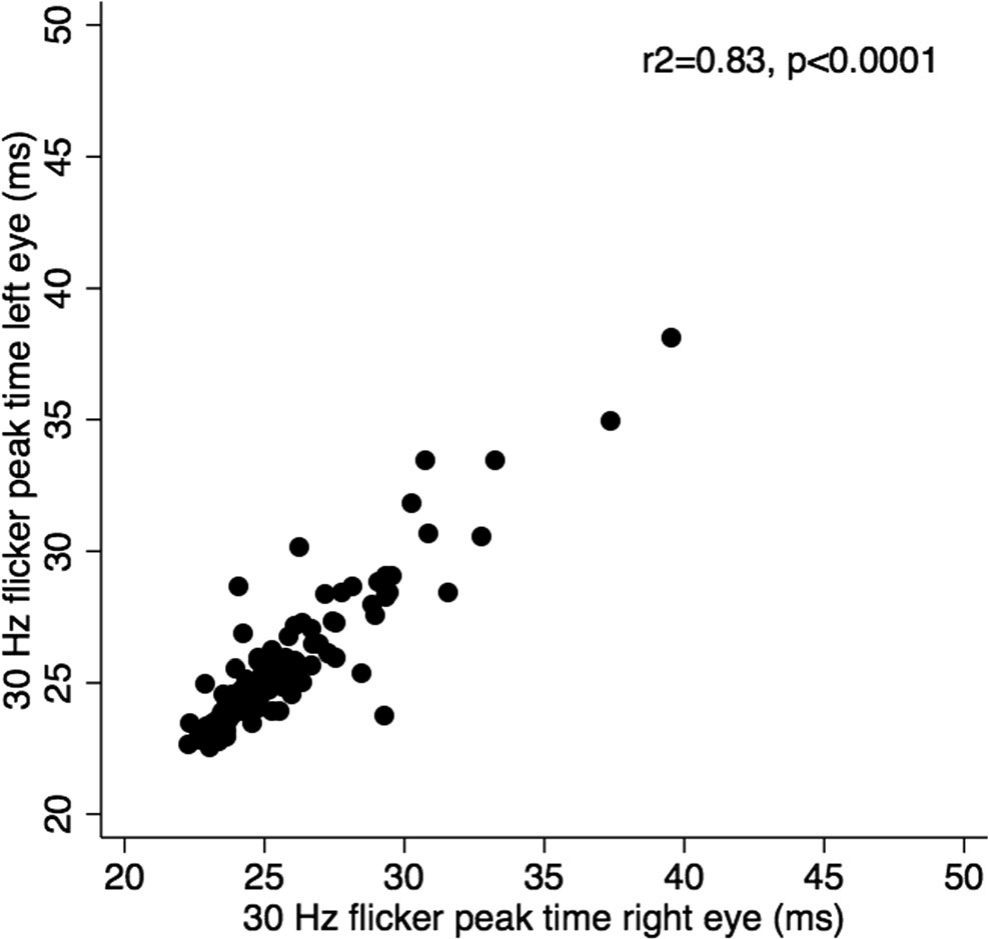
Scatter plot showing the high correlation between the 30-Hz flicker peak time measured in the right and left eyes of each subject (r^2^ = 0.83, *p* < 0.0001)

A total of 151 patients were examined with photopic 30-Hz flicker ERG, and photopic single-flash responses were also obtained in 124 of these patients. All ERG responses of the right and left eyes were highly correlated, as exemplified in the scatter plot of Fig. 1 showing the correlation in 30-Hz flicker peak time between right and left eyes (r^2^ = 0.83, *p* < 0.0001). As such, only the right eyes were used for further analysis in this study.

When all patients were included in the analysis, there was a negative association between retinopathy severity level and the b-wave amplitude of both the photopic single-flash and 30-Hz flicker responses. These associations remained statistically significant after adjusting for age (r^2^ = 0.43, *p* < 0.001; and r^2^ = 0.35, *p* < 0.001, respectively). There was also a gradual prolongation of the b-wave peak time with increasing severity of diabetic retinopathy in the photopic single-flash and 30-Hz flicker ERG, which maintained statistical significance after adjusting for age (r^2^ = 0.22, *p* = 0.009; and r^2^ = 0.48, *p* < 0.001, respectively).

**Fig. 2:**
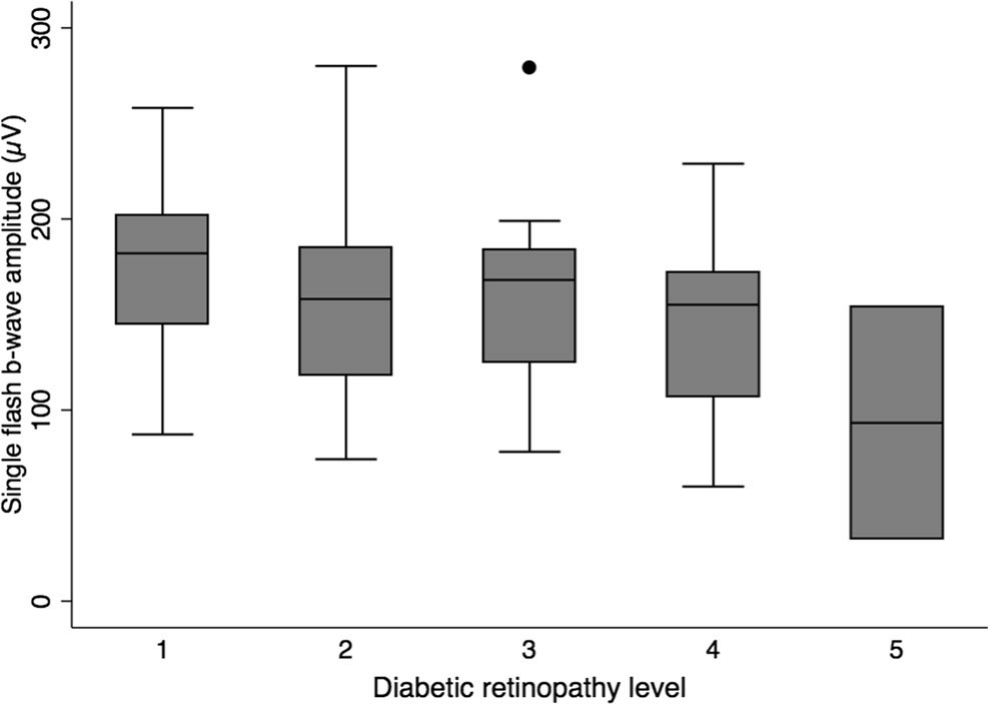
Box plot of the single-flash b-wave amplitude grouped according to the level of diabetic retinopathy, excluding laser-treated patients. Middle line: the median; bottom and top box edges: the 25th and 75th percentiles; whiskers: the most extreme values within 1.5 interquartile ranges; dots: outliers

**Fig. 3:**
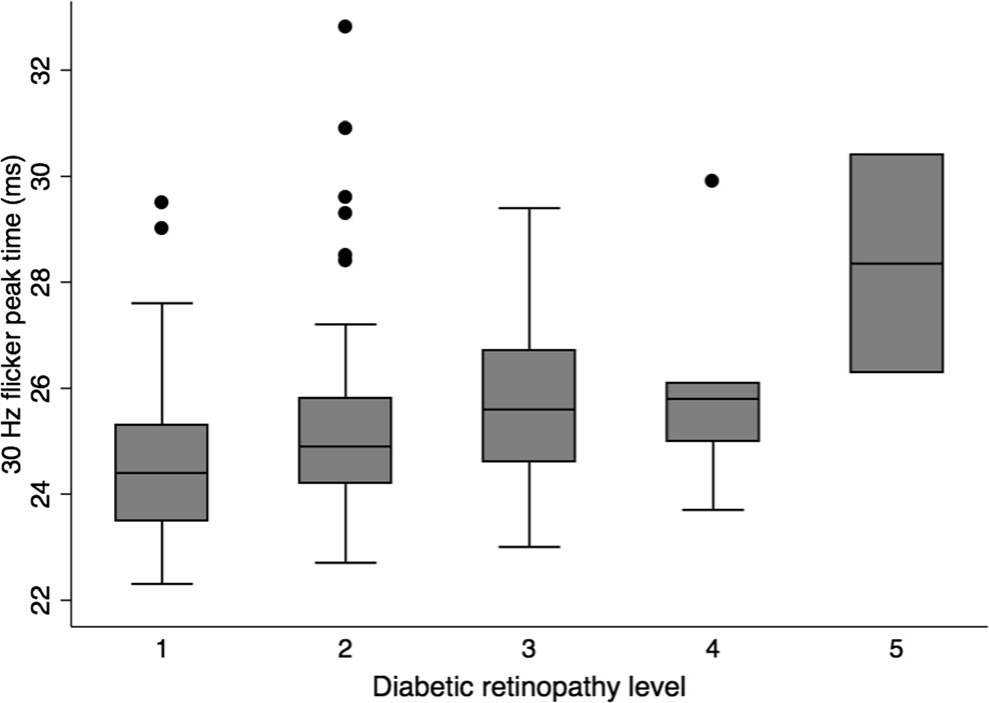
Box plot of the 30-Hz flicker peak time grouped according to the level of diabetic retinopathy, excluding laser-treated patients. Box plot details are as described in Fig. 2

When the patients who had undergone panretinal photocoagulation were excluded from the analysis (*n* = 19), there was a similar trend towards decreasing amplitudes and increasing peak time with more advanced retinopathy. For patients in the 20–50-year age group (representing 82 % of patients), the lower limit of normal single-flash b-wave amplitude was 73.7 μV and the upper limit of normal 30-Hz flicker peak time was 28.4 ms. Box plots illustrating the association (after exclusion of laser-treated patients) between the diabetic retinopathy level and single-flash b-wave amplitude, and between the retinopathy level and 30-Hz flicker peak time, are shown in Figs. 2 and 3, respectively. After adjusting for age, these associations were not statistically significant for either the b-wave amplitude of the photopic single-flash (r^2^ = 0.19, *p* = 0.59) and 30-Hz flicker (r^2^ = 0.11, *p* = 0.82) responses or the b-wave peak time of the photopic single-flash (r^2^ = 0.13, *p* = 0.16) and 30-Hz flicker (r^2^ = 0.27, *p* = 0.32) responses. When the analyses were performed without the outliers indicated in Figs. 2 and 3, only the association between retinopathy level and 30-Hz flicker peak time became statistically significant (r^2^ = 0.35, *p* = 0.03). All other associations remained non-significant.

Mean ERG parameters and standard deviations for all patients and within each group of patients at a given retinopathy level are listed in Table 2.

**Table 2:** ERG results and SD-OCT findings according to the severity level of diabetic retinopathy. All data are presented as mean ± standard deviation (SD), with the number of patients (*n*) in parentheses

Variable	All patients	Patients grouped by level of diabetic retinopathy				
		1	2	3	4	5
Single-flash ERG, b-wave amplitude, μV	149.1 ± 59.8 (*n* = 124)	174.9 ± 42.8 (*n* = 46)	158.5 ± 54.0 (*n* = 33)	155.6 ± 46.0 (*n* = 20)	131.4 ± 65.9 (*n* = 7)	65.3 ± 45.5 (*n* = 18)
Single-flash ERG, b-wave peak time, ms	31.2 ± 2.0 (*n* = 124)	30.6 ± 1.2 (*n* = 46)	30.8 ± 1.1 (*n* = 33)	31.2 ± 1.1 (*n* = 20)	30.0 ± 2.0 (*n* = 7)	33.1 ± 4.0 (*n* = 18)
30-Hz flicker ERG, amplitude, μV	61.0 ± 27.3 (*n* = 151)	70.6 ± 24.4 (*n* = 55)	65.9 ± 23.3 (*n* = 45)	62.2 ± 20.9 (*n* = 24)	58.5 ± 23.5 (*n* = 7)	22.9 ± 19.2 (*n* = 20)
30-Hz flicker ERG, peak time, ms	25.9 ± 3.0 (*n* = 151)	24.6 ± 1.6 (*n* = 55)	25.4 ± 2.1 (*n* = 45)	25.7 ± 1.7 (*n* = 24)	25.6 ± 2.0 (*n* = 7)	30.6 ± 4.5 (*n* = 20)
Central foveal thickness, μm	212.1 ± 30.1 (*n* = 151)	206.2 ± 27.8 (*n* = 55)	215.3 ± 27.9 (*n* = 45)	213.4 ± 31.1 (*n* = 24)	207.7 ± 27.3 (*n* = 7)	221.2 ± 39.3 (*n* = 20)
Mean retinal thickness, μm	265.8 ± 19.0 (*n* = 151)	266.2 ± 15.4 (*n* = 55)	264.3 ± 14.4 (*n* = 45)	268.9 ± 17.6 (*n* = 24)	266.8 ± 57.1 (*n* = 7)	264.5 ± 34.3 (*n* = 20)
Total macular volume, mm^3^	7.5 ± 0.5 (*n* = 151)	7.5 ± 0.4 (*n* = 55)	7.5 ± 0.4 (*n* = 45)	7.6 ± 0.5 (*n* = 24)	7.5 ± 0.7 (*n* = 7)	7.5 ± 1.0 (*n* = 20)

### Association between SD-OCT parameters and level of diabetic retinopathy

All 151 patients were examined using SD-OCT. As the mean retinal thickness and mean macular volume were almost perfectly correlated (r^2^ = 0.99, *p* < 0.001), we decided to focus on the central foveal thickness and mean retinal thickness, as described in the following analysis. Similar to the ERG data, the central foveal thickness and mean retinal thickness in the right and left eyes of patients showed highly significant correlations (r^2^ = 0.45, *p* < 0.001; and r^2^ = 0.71, *p* < 0.001, respectively), and we therefore used only the SD-OCT data obtained from the right eye of all subjects for further analysis.

**Fig. 4:**
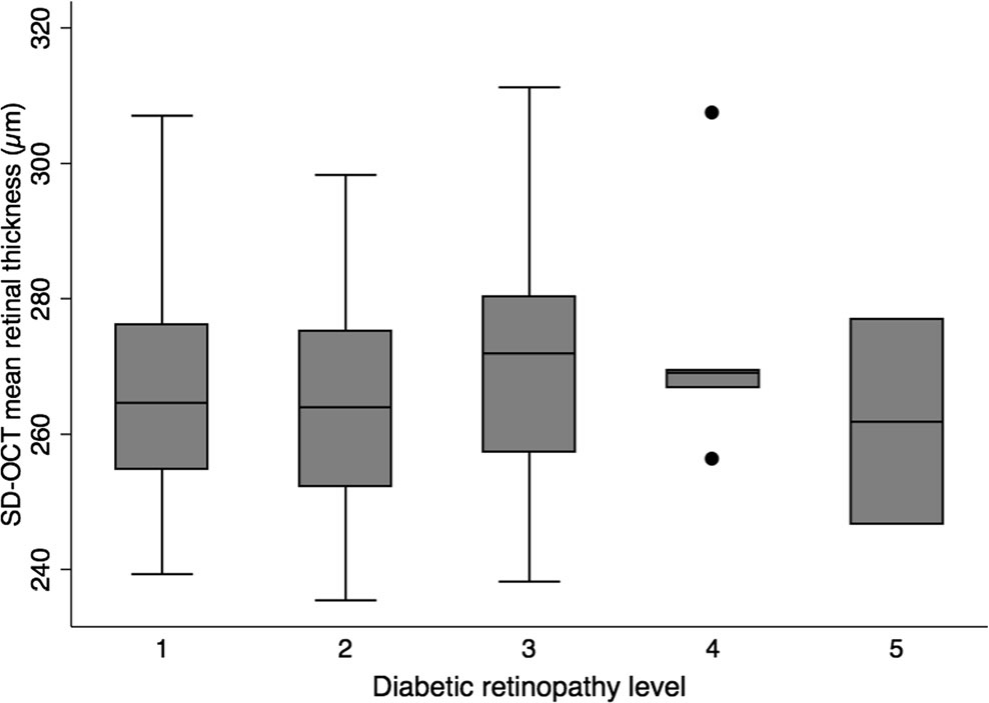
Box plot of the mean retinal thickness grouped according to the level of diabetic retinopathy, excluding laser-treated patients. Box plot details are as described in Fig. 2

There was no statistically significant association between retinopathy severity levels and retinal thickness parameters, regardless of whether the data were age-adjusted. After adjusting for age, no significant association was found between the level of retinopathy and central foveal thickness or mean retinal thickness, either when the laser-treated patients were included in the analysis (r^2^ = 0.03, *p* = 0.54; and r^2^ = 0.03, *p* = 0.79, respectively) or when they were excluded (r^2^ = 0.03, *p* = 0.63; and r^2^ = 0.03, *p* = 0.48, respectively). Similarly, excluding the nine patients with macular oedema from the analysis did not significantly affect these results. The relationship between the retinopathy severity level and mean retinal thickness (when laser-treated patients are excluded) is illustrated in Fig. 4. Mean SD-OCT parameters and standard deviations for all patients and within each group of patients at a given retinopathy level are listed in Table 2.

### Association between ERG and SD-OCT parameters

**Fig. 5:**
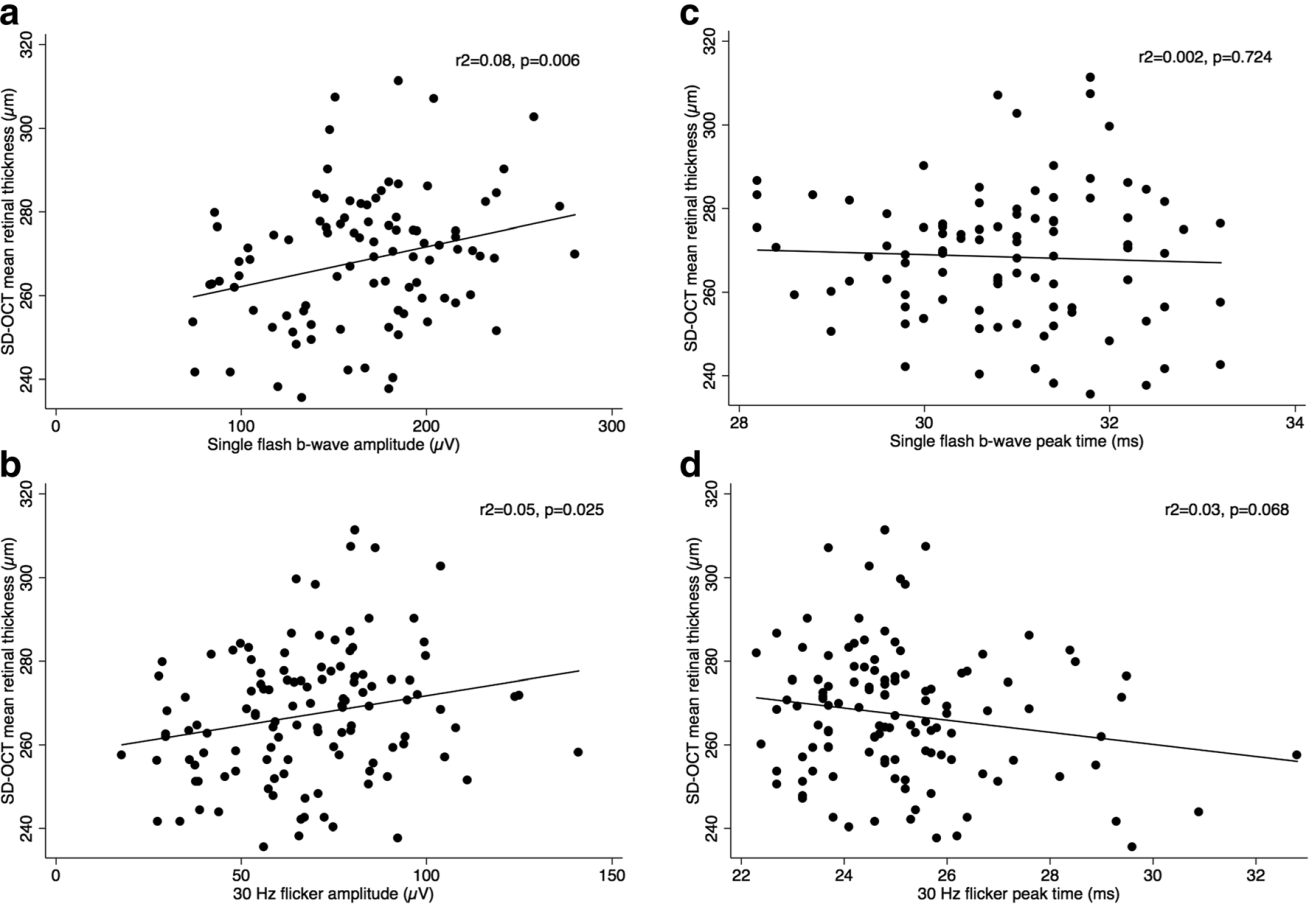
**a**–**d** Scatter plots (excluding laser-treated patients) of mean retinal thickness as a function of **a** the single-flash b-wave amplitude (r^2^ = 0.08, *p* = 0.006), **b** the 30-Hz flicker amplitude (r^2^ = 0.05, *p* = 0.025), **c** the single-flash b-wave peak time (r^2^ = 0.002, *p* = 0.724), and **d** the 30-Hz flicker peak time (r^2^ = 0.03, *p* = 0.068). The fitted regressions are shown as solid lines

Among patients who underwent both ERG and SD-OCT examinations, and after age adjustment and exclusion of those who had received panretinal photocoagulation, there was a significant association between SD-OCT-derived mean retinal thickness and the b-wave amplitudes of photopic single-flash and 30-Hz flicker responses (r^2^ = 0.08, *p* = 0.006; and r^2^ = 0.05, *p* = 0.025, respectively). There was also a gradual prolongation of the peak time with reduction of retinal thickness, indicating a weak negative association between the mean retinal thickness and the peak time of the 30-Hz flicker responses (r^2^ = 0.03, *p* = 0.068). There was no association between the mean retinal thickness and peak time of the single-flash responses (r^2^ = 0.002, *p* = 0.724) or between the central foveal thickness and any of the ERG parameters. Scatter plots illustrating the associations between mean retinal thickness and the various ERG parameters are shown in Fig. 5.

## Discussion

The gold standard for the detection and grading of diabetic retinopathy has traditionally been retinal examination by indirect ophthalmoscopy or 7-field stereoscopic photographs of each eye as defined by the ETDRS [29]. In light of current concepts of neurodegeneration in the pathogenesis of diabetic retinopathy, additional diagnostic tools like ERG and SD-OCT may also be of value in the management of the disease. In the present study, we investigated possible associations between photographically determined retinopathy levels, various photopic ERG parameters, and central retinal thickness measurements assessed by SD-OCT. As would be expected in a population-based analysis of diabetes complications, there were substantial variations in age, diabetes duration, and sample size among the five levels of disease severity. The distribution of retinopathy severity levels and the frequency of macular oedema among patients largely corresponded with that found in other studies [32–34].

The patients with macular oedema had greater central foveal thickness compared to the rest of the study population. There was no significant difference in mean retinal thickness, which may have been due to the fact that the oedema in most cases presented as a small area of focal retinal thickening in the parafoveal or extrafoveal regions. Macular oedema was determined from stereoscopic fundus photographs and was based on the presence of retinal thickening or hard exudates in the posterior pole [4]. This method is not without limitations [35], and as such, some patients with macular oedema may have been missed. While ophthalmoscopy or stereoscopic fundus photography is still recommended for routine diagnosis of diabetic macular oedema, the use of SD-OCT is increasing, and it is regarded by many as the new reference standard [35].

In order to determine the correct stage and to detect the most subtle changes in diabetic retinopathy, such as the first appearance of microaneurysms, intraretinal microvascular abnormalities (IRMA), or neovascularization, the stereoscopic colour photographs were compared to the red-free fundus images [36]. This approach of analysing both colour and red-free fundus photographs based on the International Clinical Diabetic Retinopathy Disease Severity Scale [4] led to a high degree of interobserver agreement in the assessment of retinopathy severity level.

The primary analysis of the cohort showed a clear trend of decreasing b-wave amplitude and increasing 30-Hz flicker peak time with the progression of retinopathy, and these associations remained statistically significant after age adjustment. Both the prevalence and severity of diabetic retinopathy correlate with age [37], and previous studies have shown that amplitudes decline and peak times increase with ageing [38, 39]. Although the ageing effect on these ERG parameters is relatively small, it must be taken into account, as patient age and severity of diabetic retinopathy are so closely interrelated. In addition, laser treatment has an attenuating effect on ERG parameters. Previous studies have shown a marked reduction in the amplitude of both a- and b-waves and a delay in b-wave peak time after panretinal laser treatment [17, 40, 41]. Thus, ageing and laser treatment have a synergistic negative influence on ERG response. When the patients who had previously undergone panretinal photocoagulation were excluded from the analyses, the age-adjusted associations between retinopathy level and ERG results were no longer statistically significant. Some of the earlier studies that reported significant full-field ERG changes in diabetes patients had included the additional performance of fluorescein angiography [16–18], which may have the advantage of more precise retinopathy staging. In the present study, however, we analysed a large population-based cohort of randomly selected patients with type 1 diabetes and performed detailed age-adjusted ERG analyses among five severity levels of diabetic retinopathy, and we believe that these differences in patient selection and statistical methods contributed to the discrepancy between our findings and those that have been reported by others.

We found no obvious association between the severity of diabetic retinopathy and any of the SD-OCT-derived retinal thickness measurements. The morphological parameters remained stable across all retinopathy levels regardless of whether the laser-treated patients were included in the analysis. Panretinal photocoagulation, however, has been shown to induce retinal thickening in both the peripapillary and macular regions, at least during the first year after treatment [42]. Several studies have demonstrated decreased retinal thickness with age [43–45]. Similar to what has been found in early stages of diabetic retinopathy [7], Ooto et al. showed a selective loss of inner-layer thickness with ageing [44], while Sung et al. found stable foveal thickness despite general retinal thinning with advancing age [45]. Although direct comparisons of our data with those of other studies are difficult given the different population characteristics and the use of different optical coherence tomography systems, the retinal thickness parameters were within normal population ranges [46, 47]. Even without age adjustment, we found no significant association between retinopathy level and retinal thickness. This lack of association, coupled with the conflicting results in the literature, suggests that current SD-OCT capabilities with regard to clinical staging of diabetic retinopathy are rather limited, except in the assessment of diabetic macular oedema.

To the best of our knowledge, this is the first study to report on the relationship between full-field ERG parameters and changes in retinal thickness as measured by optical coherence tomography in patients with diabetes. We found a statistically significant association between mean retinal thickness and the b-wave amplitude of photopic single-flash and 30-Hz flicker responses. This was a somewhat unexpected finding, since there was no clear association between the level of diabetic retinopathy and any of the retinal thickness parameters. However, as noted by Oshitari et al. [26], in the early stages of diabetic retinopathy, thinning of the inner retina may not be detected by optical coherence tomography measurements because increased vascular leakage may mask the effects of neuronal degeneration. Thus, while SD-OCT may also reflect functional loss due to neurodegeneration, its usefulness in the staging of diabetic retinopathy is limited due to the vascular hyperpermeability, which probably develops in parallel with the neurodegenerative process.

Limitations of the study include its cross-sectional design, the relatively small number of participants with severe non-proliferative and untreated proliferative retinopathy, and the lack of a non-diabetic age-matched control group. Furthermore, only total retinal thickness was measured, and the level of glycemic control and the presence of combined systemic diseases were not included in the analyses. One could also argue that it would be more appropriate to use multifocal ERG rather than full-field ERG to investigate the association between functional and morphological parameters, as this technique provides a more detailed mapping of the central retina and has been shown to correlate with the development of diabetic retinopathy [48, 49]. On the other hand, full-field ERG measures the summed response from the entire retina, and although we have shown that ischemic vascular changes are preferentially located in the posterior pole and along the vascular arcades [50], the diabetic vasculopathy and neuronal damage may well lead to a generalized dysfunction of the retina. Moreover, the procedure for performing full-field ERG is less time-consuming and the required equipment more readily available and affordable than for multifocal ERG.

In conclusion, we found no significant association between the severity of diabetic retinopathy and photopic ERG parameters among patients who were naïve to laser treatment. SD-OCT-derived retinal thickness measurements in the macular area did not correlate with the severity of retinopathy, but revealed a significant association with the b-wave amplitude of photopic single-flash and 30-Hz flicker responses. Given the wide diversity in the results within each diabetic retinopathy level, we believe that both full-field ERG and central retinal thickness measurements have limited clinical value in the staging of retinopathy in unselected diabetes patients. However, thinning of retinal tissue leads to significant functional impairment, and may reflect an ongoing neurodegenerative process of the retina. Ideally, diagnostic tools like ERG and SD-OCT should be able to identify patients who are most at risk of developing proliferative retinopathy so that closer follow-up and timely treatment can be offered. This needs to be explored in larger longitudinal studies, which are clearly warranted.

## References

[1] Wild S, Roglic G, Green A, Sicree R, King H (2004). Global prevalence of diabetes: estimates for the year 2000 and projections for 2030. Diabetes Care.

[2] Klein R, Klein BE, Moss SE, Davis MD, DeMets DL (1989). The Wisconsin epidemiological study of diabetic retinopathy. IX. Four-year incidence and progression of diabetic retinopathy when age of diagnosis is less than 30 years. Arch Ophthalmol.

[3] Early Treatment Diabetic Retinopathy Study Research Group (1991). Early photocoagulation for diabetic retinopathy. ETDRS report number 9. Ophthalmology.

[4] Wilkinson CP, Ferris FL, Klein RE (2003). Proposed international clinical diabetic retinopathy and diabetic macular edema disease severity scales. Ophthalmology.

[5] Barber AJ, Lieth E, Khin SA, Antonetti DA, Buchanan AG, Gardner TW (1998). Neural apoptosis in the retina during experimental and human diabetes. J Clin Invest.

[6] Abu El-Asrar AM, Dralands L, Missotten L, Al-Jadaan IA, Geboes K (2004). Expression of apoptosis markers in the retinas of human subjects with diabetes. Invest Ophthalmol Vis Sci.

[7] van Dijk HW, Kok PH, Garvin M (2009). Selective loss of inner retinal layer thickness in type 1 diabetic patients with minimal diabetic retinopathy. Invest Ophthalmol Vis Sci.

[8] Antonetti DA, Klein R, Gardner TW (2012). Diabetic retinopathy. N Engl J Med.

[9] Scholl HP, Zrenner E (2000). Electrophysiology in the investigation of acquired retinal disorders. Surv Ophthalmol.

[10] Marmor MF, Holder GE, Seeliger MW, Yamamoto S, International Society for Clinical Electrophysiology of Vision (2004). Standard for clinical electroretinography (2004 update). Doc Ophthalmol.

[11] Tzekov R, Arden GB (1999). The electroretinogram in diabetic retinopathy. Surv Ophthalmol.

[12] Yonemura D, Aoki T, Tsuzuki K (1962). Electroretinogram in diabetic retinopathy. Arch Ophthalmol.

[13] Gjötterberg M (1974). The electroretinogram in diabetic retinopathy. A clinical study and a critical survey. Acta Ophthalmol (Copenh).

[14] Simonsen SE (1974). Prognostic value of ERG (oscillatory potential) in juvenile diabetics. Acta Ophthalmol Suppl.

[15] Bresnick GH, Palta M (1987). Oscillatory potential amplitudes. Relation to severity of diabetic retinopathy. Arch Ophthalmol.

[16] Chung NH, Kim SH, Kwak MS (1993). The electroretinogram sensitivity in patients with diabetes. Korean J Ophthalmol.

[17] Holopigian K, Seiple W, Lorenzo M, Carr R (1992). A Comparison of photopic and scotopic electroretinographic changes in early diabetic retinopathy. Invest Ophthalmol Vis Sci.

[18] Bresnick GH, Palta M (1987). Temporal aspects of the electroretinogram in diabetic retinopathy. Arch Ophthalmol.

[19] Satoh S, Iijima H, Imai M, Abe K, Shibuya T (1994). Photopic electroretinogram implicit time in diabetic retinopathy. Jpn J Ophthalmol.

[20] Lattanzio R, Brancato R, Pierro L (2002). Macular thickness measured by optical coherence tomography (OCT) in diabetic patients. Eur J Ophthalmol.

[21] Sánchez-Tocino H, Alvarez-Vidal A, Maldonado MJ, Moreno-Montañés J, García-Layana A (2002). Retinal thickness study with optical coherence tomography in patients with diabetes. Invest Ophthalmol Vis Sci.

[22] Goebel W, Franke R (2006). Retinal thickness in diabetic retinopathy: comparison of optical coherence tomography, the retinal thickness analyzer, and fundus photography. Retina.

[23] Sng CC, Cheung CY, Man RE (2012). Influence of diabetes on macular thickness measured using optical coherence tomography: the Singapore Indian Eye Study. Eye.

[24] Biallosterski C, van Velthoven ME, Michels RP, Schlingemann RO, DeVries JH, Verbraak FD (2007). Decreased optical coherence tomography-measured pericentral retinal thickness in patients with diabetes mellitus type 1 with minimal diabetic retinopathy. Br J Ophthalmol.

[25] Asefzadeh B, Fisch BM, Parenteau CE, Cavallerano AA (2008). Macular thickness and systemic markers for diabetes in individuals with no or mild diabetic retinopathy. Clin Exp Ophthalmol.

[26] Oshitari T, Hanawa K, Adachi-Usami E (2009). Changes of macular and RNFL thicknesses measured by Stratus OCT in patients with early stage diabetes. Eye.

[27] Verma A, Rani PK, Raman R (2009). Is neuronal dysfunction an early sign of diabetic retinopathy? Microperimetry and spectral domain optical coherence tomography (SD-OCT) study in individuals with diabetes, but no diabetic retinopathy. Eye.

[28] van Dijk HW, Verbraak FD, Kok PH (2010). Decreased retinal ganglion cell layer thickness in patients with type 1 diabetes. Invest Ophthalmol Vis Sci.

[29] Early Treatment Diabetic Retinopathy Study Research Group (1991). Grading diabetic retinopathy from stereoscopic color fundus photographs—an extension of the modified Airlie House classification. ETDRS report number 10. Ophthalmology.

[30] McCulloch DL, Marmor MF, Brigell MG (2015). ISCEV Standard for full-field clinical electroretinography (2015 update). Doc Ophthalmol.

[31] Early Treatment Diabetic Retinopathy Study research group (1985). Photocoagulation for diabetic macular edema. Early Treatment Diabetic Retinopathy Study report number 1. Arch Ophthalmol.

[32] Sivaprasad S, Gupta B, Crosby-Nwaobi R, Evans J (2012). Prevalence of diabetic retinopathy in various ethnic groups: a worldwide perspective. Surv Ophthalmol.

[33] Sivaprasad S, Gupta B, Gulliford MC (2012). Ethnic variations in the prevalence of diabetic retinopathy in people with diabetes attending screening in the United Kingdom (DRIVE UK). PLoS One.

[34] Knudsen LL, Lervang HH, Lundbye-Christensen S, Gorst-Rasmussen A (2006). The North Jutland County Diabetic Retinopathy Study: population characteristics. Br J Ophthalmol.

[35] Virgili G, Menchini F, Casazza G (2015). Optical coherence tomography (OCT) for detection of macular oedema in patients with diabetic retinopathy. Cochrane Database Syst Rev.

[36] Bernardes R, Serranho P, Lobo C (2011). Digital ocular fundus imaging: a review. Ophthalmologica.

[37] Klein R, Klein BE, Moss SE, Davis MD, DeMets DL (1984). The Wisconsin epidemiologic study of diabetic retinopathy. II. Prevalence and risk of diabetic retinopathy when age at diagnosis is less than 30 years. Arch Ophthalmol.

[38] Weleber RG (1981). The effect of age on human cone and rod ganzfeld electroretinograms. Invest Ophthalmol Vis Sci.

[39] Wright CE, Williams DE, Drasdo N, Harding GF (1985). The influence of age on the electroretinogram and visual evoked potential. Doc Ophthalmol.

[40] Perlman I, Gdal-On M, Miller B, Zonis S (1985). Retinal function of the diabetic retina after argon laser photocoagulation assessed electroretinographically. Br J Ophthalmol.

[41] Capoferri C, Bagini M, Chizzoli A, Pece A, Brancato R (1990). Electroretinographic findings in panretinal photocoagulation for diabetic retinopathy. A randomized study with blue-green argon and red krypton lasers. Graefes Arch Clin Exp Ophthalmol.

[42] Kim JJ, Im JC, Shin JP, Kim IT, Park DH (2014). One-year follow-up of macular ganglion cell layer and peripapillary retinal nerve fibre layer thickness changes after panretinal photocoagulation. Br J Ophthalmol.

[43] Alamouti B, Funk J (2003). Retinal thickness decreases with age: an OCT study. Br J Ophthalmol.

[44] Ooto S, Hangai M, Tomidokoro A (2011). Effects of age, sex, and axial length on the three-dimensional profile of normal macular layer structures. Invest Ophthalmol Vis Sci.

[45] Sung KR, Wollstein G, Bilonick RA (2009). Effects of age on optical coherence tomography measurements of healthy retinal nerve fiber layer, macula, and optic nerve head. Ophthalmology.

[46] Leung CK, Cheung CY, Weinreb RN (2008). Comparison of macular thickness measurements between time domain and spectral domain optical coherence tomography. Invest Ophthalmol Vis Sci.

[47] Wexler A, Sand T, Elsås TB (2010). Macular thickness measurements in healthy Norwegian volunteers: an optical coherence tomography study. BMC Ophthalmol.

[48] Bearse MA, Adams AJ, Han Y (2006). A multifocal electroretinogram model predicting the development of diabetic retinopathy. Prog Retin Eye Res.

[49] Lung JC, Swann PG, Wong DS, Chan HH (2012). Global flash multifocal electroretinogram: early detection of local functional changes and its correlations with optical coherence tomography and visual field tests in diabetic eyes. Doc Ophthalmol.

[50] Jansson RW, Frøystein T, Krohn J (2012). Topographical distribution of retinal and optic disc neovascularization in early stages of proliferative diabetic retinopathy. Invest Ophthalmol Vis Sci.

